# A CNN-Based Model of Cross-Immunity to Influenza A(H3N2) Virus: Testing Under “Real-World” Conditions

**DOI:** 10.3390/v18030327

**Published:** 2026-03-06

**Authors:** Marina N. Asatryan, Vaagn G. Agasaryan, Boris I. Timofeev, Ilya S. Shmyr, Dmitrii N. Shcherbinin, Elita R. Gerasimuk, Tatiana A. Timofeeva, Ivan F. Ershov, Tatiana A. Semenenko, Denis Yu. Logunov, Alexander L. Gintsburg

**Affiliations:** National Research Center for Epidemiology and Microbiology Named After Honorary Academician N.F. Gamaleya, Moscow 123098, Russia

**Keywords:** influenza A(H3N2), antigenic prediction, cross-immunity model, convolutional neural network (CNN), hemagglutination inhibition (HAI) assay, vaccine strain selection, epidemiological model

## Abstract

A cross-immunity model for influenza A(H3N2) based on convolutional neural networks (CNNs) was developed and validated under temporally structured conditions that mimic real-world forecasting. Antigenic distance was derived from hemagglutination inhibition (HI) titers. The model was trained on WHO data (2011–2023) and tested in a time-split fashion on independent recent data (2022–2024). Hemagglutinin sequences (HA/HA1) were encoded into 3D tensors using five physicochemical indices from AAindex. Two- and three-layer CNN architectures were tested. Performance was evaluated using Accuracy, Sensitivity, Specificity, and Matthews Correlation Coefficient (MCC) with 95% confidence intervals. Validation on the classic Smith’s dataset showed high accuracy (Accuracy = 0.9996, MCC = 0.9964), serving as a necessary sanity check. Testing on current data yielded lower but robust results (Accuracy: 0.73–0.81, MCC: 0.48–0.60), reflecting real-world forecasting complexity. ROC analysis confirmed the strong discriminative ability (AUC ≥ 0.805) and good calibration (Brier scores ≤ 0.192). The three-layer CNN demonstrated greater robustness on challenging data. This CNN model is an effective tool for assessing influenza A(H3N2) antigenic distances and holds promise for integration into epidemiological models to aid vaccine strain selection. Further accuracy improvements may arise from modeling the structural impact of amino acid substitutions and polyclonal immune responses.

## 1. Introduction

Influenza viruses belonging to the Orthomyxoviridae family, according to the current classification of the International Committee on Taxonomy of Viruses (ICTV—International Committee on Taxonomy of Viruses) [1], remain the most important pathogens responsible for a wide range of manifestations—from seasonal outbreaks to global pandemics. About a billion cases of seasonal influenza are recorded annually, of which 3–5 million are severe. Constantly developing research is necessary for an in-depth understanding of the pathogenesis of the virus, its genomic features, the body’s immune response to infection, as well as its epidemiological characteristics, which is an important prerequisite for the development of effective control and prevention measures [2].

A key factor for successful vaccination is a detailed characterization of circulating strains. To this end, the World Health Organization (WHO) and its partner network of national centers use traditional methods, including the hemagglutination inhibition (HAI) assay and the neutralization test, to determine the antigenic similarity between strains (WHO. World Health Organization. Available at: https://www.who.int/initiatives/global-influenza-surveillance-and-response-system (accessed on 7 February 2026)) [2,3,4,5].

The HAI assay directly measures antibody inhibition of hemagglutinin (HA) binding to sialic acids on red blood cells. Therefore, only antibodies that bind to the globular portion of HA or block sialic acid binding show a titer in the HAI assay [6].

Computational methods (in silico methods) for quantitative analysis and prediction of antigenic variants, based primarily on the concept of antigenic distance and used to predict the antigenic evolution of seasonal influenza A viruses, have recently undergone significant improvement [7,8]. Numerical approaches need to be integrated into the vaccine selection process as an independent, parallel tool, while ensuring their regular assessment of effectiveness [5,9].

A separate task in similar studies is the description of ways to represent the genetic distance and the choice of the antigenic distance. In the case of genetic distance (amino acid substitutions), this can be either a simple Hamming distance or a function of it [10], or the use of physicochemical characteristics from the AAindex database (a database of numerical indices representing various physicochemical and biochemical properties of amino acids and pairs of amino acids).

In the case of antigenic distance (antigenic difference between strains), different expressions from HAI titers are usually used [11]:

Dij = log_2_ (Hii/Hij) (1)

(2) $$\mathrm{Dij}=\log_{2}\;\sqrt{\begin{array}{c} \frac{\left(Hii*Hjj\right)}{Hij*Hji} \end{array}}$$

Archetti–Horsfall distance [12,13].

Hii, Hjj are titer values, where the serum strain acts as a test; Hij, Hji are titer values between different strains.

When using the Archetti–Horsfall distance (the distance is symmetrical with respect to both strains involved), researchers gain a certain advantage and are forced to limit the dataset to only those reactions that were carried out with serum strains.

Moreover, certain distance values may indicate the presence or absence of protection against infection with a specific influenza virus strain. In this case, the transition value is called the “antigenic escape threshold”.

To date, a wide range of methods have been used to develop models of cross-immunity: from classical statistics to machine learning methods, including complex deep learning approaches.

Thus, the works of Min-Shi Lee [14] and Wiiliam D. Lees [15] use linear and multiple linear regression, respectively, taking into account glycosylation.

With the growing volume of data and the need to extract useful knowledge from it, machine learning has become a widely used tool to solve these problems.

In particular, in Yu-Chieh Liao’s study [13], researchers use a set of methods to achieve this goal, including multiple and logistic regression, as well as a support vector machine. These methods are implemented taking into account a number of physicochemical characteristics, namely:, polarity, charge, and amino acid structure.

In the work of Jingxuan Qiu [16], a model for assessing antigenicity was developed that fully takes into account the conformational features of hemagglutinin.

In a later publication by Yousong Peng [17], the authors implemented Bayesian classification and support vector machine algorithms to solve classification and regression analysis problems.

Yuhua Yao [18] presented a new algorithm called Random Forest Joint Regression to analyze and identify the best substitution matrices. The work uses physicochemical and biochemical characteristics from the AAindex2 database.

Much of the research in this area involves the use of neural networks, which demonstrate high efficiency in solving problems of prediction, recognition, and classification. Among modern architectures, deep convolutional neural networks are of particular interest.

Convolutional neural networks (CNNs) are widely used in classification problems due to their high efficiency and accuracy. A CNN architecture includes input, hidden, and output layers. The input layer receives an input tensor representing an image and passes it to subsequent hidden layers, which may contain one or more convolutional and pooling layers for feature extraction and processing. The fully connected output layer generates a predicted class label or probability distribution over classes, enabling accurate classification of the input data [19].

In studies using neural networks, authors are involved in model development and pay special attention to data preparation.

One of the first to conduct in silico studies on the quantitative assessment of antigenic distance and prediction of the antigenicity of the influenza A virus (subtype H3N2) using convolutional neural networks (CNNs) was Eva K. Lee et al. in 2020 [20]. The researchers used data from the AAindex database, in particular the substitution matrices from AAindex2 and the numerical indicators of contact potentials from AAindex3. The authors used the definition of antigenic distance expressed by formula 1 in their calculations. To solve the problem of processing discontinuous space and selecting specific matrices from the AAindex database, the researchers applied the particle swarm optimization (PSO) algorithm.

Majid Forghani [7] also developed a method for estimating the antigenic distance for the H1N1 subtype, calculated using Formula (1), based on convolutional neural networks analyzing hemagglutinin sequences. The features are formed by taking into account the physicochemical properties of amino acids (AAindex1 matrix), optimized using the PCA (principal component analysis) method.

In the study by Rui Yin [21], a two-dimensional convolutional neural network (IAV-CNN) model was presented for predicting antigenic variants of several influenza A subtypes (H1N1, H3N2, and H5N1), where the antigenic distance is calculated using the Archetti–Horsfall formula (Equation (2)), and the ProtVec method, which takes into account spatial and channel characteristics, is used to extract significant regions of amino acid residues.

In a later work [22], Jing Meng et al. designed PREDAC-CNN with a spatially oriented representation of the HA1 (HA subunit 1) sequence for the convolutional CNN architecture. The model uses the Archetti–Horsfall distance (Equation (2)) and focuses on the physicochemical characteristics from AAindex1 that are crucial for determining the antigenicity of influenza viruses, with a preliminary biological rationale for its choice.

In addition to CNN, researchers use other neural network architectures, including linguistic models such as BLSTM bidirectional long-short-term memory, BERT, and others, as well as various combinations of them.

In Yuan-Ling Xia’s work [23], antigenic variability of the H3N2 influenza A virus is predicted using a combined CNN-BiLSTM architecture. CNN is used to analyze local patterns in amino acid sequences, while BiLSTM takes into account their long-term contextual dependencies to predict antigenic properties. The researchers trained and tested the model on the Smith dataset, using the Archetti–Horsfall equation as the antigenic distance.

In the paper by Yiming Du [24], the authors propose a hybrid DPCIPI model, the architecture of which was developed based on the BERT model, but modified by incorporating mechanisms similar to BiLSTM into its core.

One of the most complex recently published papers is that by Qitao Jia [9]. The key innovation of the study is a two-stage approach that combines both the use of a graph neural network (GNN) and the application of meta-learning to solve the problem of predicting new strains with limited data. The authors report that this approach appears to be particularly practical and effective in real-world settings.

When analyzing the results obtained by researchers using various methods and approaches, the problem arises of choosing a method and criteria for their comparison. For this purpose, many researchers use the standard Smith dataset [8] as an initial dataset, consisting of 273 influenza virus strains and 79 sera, and 4215 HAI assay results. In his work, Derek J. Smith, using the multidimensional scaling (MDS) method, plots this data on a map, thereby combining them into 11 antigenic clusters. In this case, pairs belonging to the same cluster are considered antigenically similar, and pairs belonging to different clusters are antigenically different. Researchers also use different versions of the quasi-experimental Smith’s dataset, compiled on the basis of a similar assumption and used both in the initial version (31,878 pairs of strains) and after certain filtration [18,23,25,26,27]. This allows for the comparison of the obtained results. At the same time, the methods can be different from statistical and machine learning methods to convolutional and linguistic models and their combinations.

Summarizing the research in this area, it can be concluded that researchers primarily use HAI assay data starting from 1968 and most often employ the Archetti–Horsfall distance. It should be noted, however, that the further back the data goes, the more frequently noticeably sparse and incorrect records are encountered, and the model’s ability to detect finer differences is lost. This obscures significant nuances over the retrospective period. It is also important to note that in the studies presented above, as in the present study, the authors assume that sera induced by different isolates but based on identical sequences are immunologically equivalent and interchangeable within the model.

**Figure 1:**
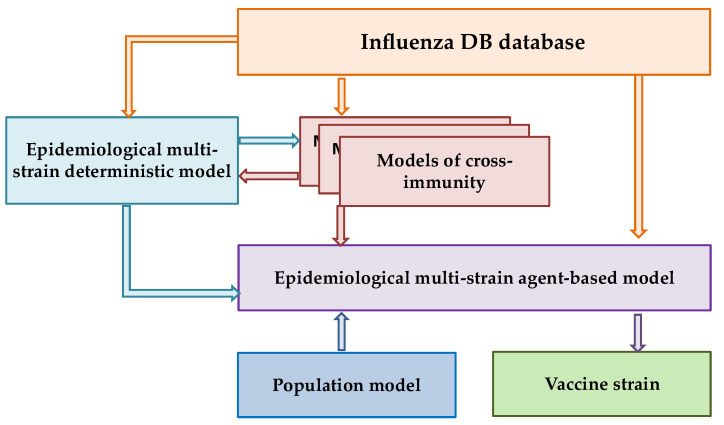
Integrated Influenza IDE model.

Figure 1 shows the block diagram of the Influenza IDE computer program (an epidemiological multi-strain model (EMM) with a cross-immunity model and a constantly updated database (of various types and subtypes of the influenza virus, Influenza DB), developed by the team of the Gamaleya Center [28].

The ultimate goal of our research program is to select a vaccine strain using a validated epidemiological multi-strain agent-based model (Influenza IDE). This approach aims to verify the immune landscape in the population and predict the prevalence of individual strains in the upcoming season. The present study focuses on an important intermediate step within this broader framework: the development and rigorous validation of a cross-immunity model.

The core conceptual innovation of our study lies in aligning the validation strategy with the model’s intended operational use. Specifically, we assess the model’s ability to perform antigenic classification under conditions that closely mimic a real forecasting task. To this end, we introduce a temporally structured validation protocol that explicitly mimics real-world forecasting: the model is trained on data up to a given season and tested exclusively on subsequent, non-overlapping seasons. This approach is not merely a technical refinement but a fundamental prerequisite for the model’s ultimate purpose—integration into the Influenza IDE epidemiological framework to simulate strain competition and inform vaccine strain selection in silico.

Since the cross-immunity model is the central focus of the computer program, Influenza IDE is designed to integrate various cross-immunity models. This potential allows for further customization and optimization of both the cross-immunity model itself and subsequent verification of the immune landscape [10,28].

## 2. Materials and Methods

In this study, we validate the model using a temporally structured cross-validation scheme designed to approximate the conditions of a real-world forecasting task. Our approach focuses on antigenic classification and is characterized by the following:

(1) The use of modern HAI assay data (2011–2024) obtained after the introduction of neuraminidase inhibitors (oseltamivir) into practice, ensuring relevance to the current antigenic landscape;

(2) Calculating the antigenic distance using Equation (3) (see below), which allows us to utilize the entire dataset without limiting it to symmetric pairs, thereby maximizing the available information;

(3) A rigorous time-split testing procedure, where the model is trained on data up to a given season, and its predictive ability is assessed exclusively on data from subsequent, non-overlapping seasons.

At the same time, to correctly compare the results of our model with the works of other researchers, we also carried out computational experiments with the quasi-experimental Smith dataset, accordingly using the antigenic distance calculated with the Archetti–Horsfall equation at this stage (Equation (2)).

### 2.1. Selection of Antigen Distance

Our model uses an expression from HI titers as a measure of cross-immunity. For this, published titer values are converted using Formula (3):

Dij = log_2_(Hi_max/Hij) (3)

where H i_max is the titer value, where the test value in our study is the maximum titer value in experiments with a specific reference strain, and Hij is the titer between different strains. In this study, the antigenic escape threshold was determined to be Dij equal to 2 log unit (Dij = 2).

The choice of antigenic distance definition was guided by the need to maximize the use of available HAI data. While the Archetti–Horsfall distance (Equation (2)) provides a symmetric measure that accounts for reciprocal titers, its application requires the availability of both serum strains, which substantially reduces the number of usable pairs. In contrast, the distance defined in Equation (3) can be computed for any pair where the test strain has been tested against a given serum, regardless of whether the reciprocal measurement exists. This allows us to retain the full richness of the WHO dataset, including all 110,679 HAI reactions.

Moreover, this formulation has a clear biological interpretation and follows a well-established precedent in the field. Hi_max represents the homologous titer for the serum strain, and Dij quantifies the fold-reduction in titer relative to this reference. The threshold Dij ≥ 2 therefore corresponds to a four-fold or greater reduction, a widely used criterion for antigenic difference in influenza surveillance. This approach is consistent with the logic underlying classical antigenic cartography methods [8] and subsequent models of influenza antigenic evolution [29], which also rely on log-transformed titer ratios with homologous references.

Importantly, this threshold is not an optimized parameter derived from our data but a biologically grounded standard adopted from the literature. The sensitivity analysis presented below serves to demonstrate that model performance remains stable around this conventional value, rather than to identify an ‘optimal’ threshold for our specific dataset.

### 2.2. Data Preparation

The results of serum testing for hemagglutination inhibition, covering reference and test strains of influenza A(H3N2) virus, were taken from the WHO’s published seasonal reports [30].

Since the WHO in 2008–2009 discovered a significant influence of neuraminidase and erythrocytes from various objects (turkey, human, guinea pig) on the HAI assay results, further studies used and processed only reports from 2011 to 2024, performed on guinea pig erythrocytes with the addition of 20 nM Oseltamivir, which is used to exclude the influence of neuraminidase. Meanwhile, these data also contain strains from an earlier period, starting from 2007. The number of HAI reactions performed, presented in the reports, is 110,679.

Virus sequence records with accompanying information were obtained from the GISAID (Global Initiative on Sharing All Influenza Data) platform [31]. GISAID includes genetic sequence and related clinical and epidemiological data. After cleaning and reconciling the data from GISAID and subsequent alignment to the reference sequence, a table with complete sequences was generated.

Matching strain names between WHO reports and GISAID sequences was performed using a semi-automated C++ module (ISO C++ 17 Standard, IDE Visual Studio 2019 CE) with case-insensitive comparison, handling of punctuation and geographical abbreviations. During the matching process between HAI records and GISAID sequence data, records were excluded if the strain name could not be reliably matched, if complete hemagglutinin (HA) or subunit 1 (subunit HA1) sequences were unavailable, or if sequence quality was insufficient. From the initial set of 110,679 HAI records, a total of 12,251 records were excluded due to these reasons.

For strains with multiple associated sequences in GISAID, the most frequently occurring sequence for that strain was selected. This resulted in 2227 unique sequence entries for the entire hemagglutinin sequence (HA sequence) and 1751 for subunit 1 (subunit HA1 sequence).

In cases where multiple HAI titer values were available for a pair of strains with identical HA sequences, the corresponding antigenic distances (calculated using Equation (3)) were aggregated by arithmetic averaging. Arithmetic averaging of log_2_-transformed distances is mathematically equivalent to calculating the antigenic distance from the geometric mean titer (GMT) of the original measurements. The use of GMT for summarizing replicate HAI data is a well-established practice in influenza serology, employed to reduce the impact of random assay variability [32].

Models were trained from 2011 to 2021, with one year added to the training dataset at a time. Models were tested on data from 2022 to 2024, with one year in each simulation. The number of unique pairs of complete sequences with antigenic distances is shown in Table 1 with a positive feature distribution.

### 2.3. AAindex Selection

To assess the contribution of each amino acid to the change in antigenic distance in this study, the AAindex1 matrices were used [33].

The AAindex1 database contains various physicochemical characteristics of 20 amino acids. From these, we selected those characteristics that are important in terms of antigen–antibody binding: hydrophobicity, polarity, charge, volume, and accessible surface area [22,34]. To select specific matrices corresponding to these characteristics, we used the classification available in the AAindex1 database and performed additional filtering (we excluded matrices with missing values or, for example, for charge, we removed matrices responsible only for positive and negative charges and left the matrix responsible for the net charge). As a result, the matrices from the AAindex1 list were combined into five groups, respectively: hydrophobicity (49), polarity (4), charge (1), volume (12), and accessible surface (4).

For each of these groups (except charge), a 20 rows (amino acids) * N columns (AAindex1 corresponding entries) table was constructed, which was then processed by principal component analysis (PCA) from the R statistical computing language package (version 4.2.2) to eliminate matrices that were highly dependent on others.

These calculations identified specific matrices in the corresponding groups with a variance coverage greater than 90%. Table 2 presents selected entries from AAindex1 across five physicochemical groups.

For further calculations, a combination (one specific matrix in each physicochemical group) with the best accuracy metric score is selected using a CNN. A model with two convolutional layers and a length of 329 amino acid sequences was used for this purpose (see below). Table 3 presents the final list of indices from AAindex1 that demonstrated the best value.

Thus, as input data for the CNN, for each pair of HAI assays, a three-dimensional input tensor, pattern, color image is formed with the following dimensions: in length, the number of HA positions both in 550 amino acid sequences and limited only by the subunit 1 of hemagglutinin (HA1), equal to 329 amino acid sequences; in height, two participating strains (serum and test); in depth, five layers corresponding to the selected AAindex1 matrices according to physicochemical characteristics, which represent individual colors composing the color image.

**Figure 2:**
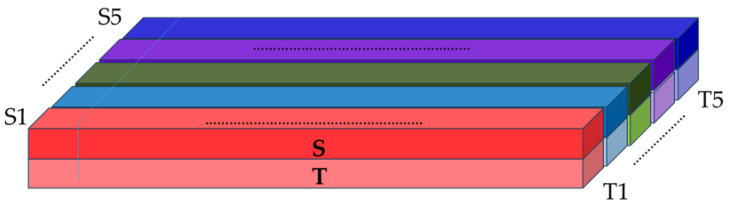
Construction of the 3D input tensor. The tensor dimensions are (L, 2, 5), where L = 329 (subunit HA1) or 550 (HA). The first dimension corresponds to amino acid positions; the second dimension encodes the serum and test strains; the five channels represent physicochemical properties (hydrophobicity, polarity, charge, volume, accessible surface area). Each cell contains the normalized value for the corresponding amino acid, strain, and property.

Accordingly, each pixel (cube) of this image in each of the five layers shows at the intersection the value of the AAindex1 category and the amino acid position of the protein site in the test or serum strain. Next, the three-dimensional input tensor is fed into the network, where each column (by multiplying the value of AAindex1 at a specific position by the corresponding coefficient, which is subsequently varied and selected by the network) turns into a combined color (represented by a number) and thus we get a flat image with new colors (Figure 2).

In the next step, we quantified the antigenic distance values using an antigenic escape threshold of 2. Thus, for each pair of sequences, we obtained a binary indicator of the presence or absence of protection. Since these binary data correspond to specific graphic patterns (colored images), the final task of this study was to classify these images (pattern recognition).

As a result, having such a trained neural network, it is possible to predict HAI assay results for an arbitrary pair of strains.

### 2.4. Neural Network Description

For the computational experiments in our study, we used CNN models with different architectures, with two and three convolutional layers. The use of a model with an additional third convolutional layer was intended to detect higher-level patterns and thereby improve the model’s representativeness.

Both architectures were designed to process the 3D input tensor representing pairs of influenza strains (serum and test) across five physicochemical channels (Figure 2). Table 4 summarizes the architectural details for the subunit HA1 model; full details for the full-length HA model are provided in Appendix B.

All models were trained with the following settings:

- Loss function: Binary cross-entropy;
- Optimizer: Adam with learning rate 1 × 10^−4^;
- Batch size: 32;
- Maximum epochs: 50;
- Early stopping: Two criteria applied: (1) stop if train–validation loss gap exceeds 0.05 after epoch 5; (2) validation loss with patience 3 and minimum improvement threshold 0.001;
- Weight initialization: Kaiming initialization for convolutional layers, Xavier initialization for linear layers;
- Reproducibility: Fixed random seeds for Python, NumPy 1.21.0 and PyTorch (CPU/CUDA); cuDNN deterministic mode enabled.

The neural network model was designed and implemented in Python version 3.12.3.

The PyTorch deep learning framework version 2.10 was chosen as the main tool for creating the network architecture, organizing the training process, and conducting experiments.

### 2.5. Metrics for Evaluating the Results Obtained

**Figure 3:**
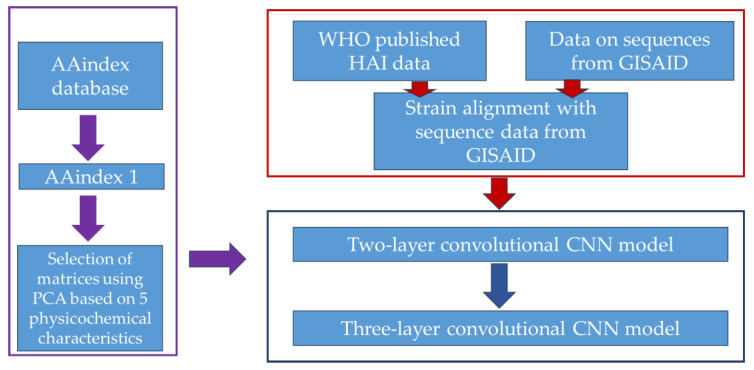
Design of a cross-immunity model.

As a measure of the adequacy (quality and accuracy of the forecast) of the models, the quality metrics adopted in binary classification problems were used: Accuracy; Sensitivity or the proportion of true positive results (true positive rate, TPR); and Specificity (the proportion of true negative results, TNR); MCC (Matthews Correlation Coefficient), a balanced measure of efficiency. The design of the cross-immunity model is shown in Figure 3.

The data block used in the work is highlighted in red. The AAindex block is highlighted in purple. The CNN model block is highlighted in dark blue.

Separately, for comparison with the results of other researchers who used the quasi-experimental Smith dataset in their calculations, we pre-trained our model on the Smith dataset from 1968 to 1998 and then tested it on the period from 1999 to 2003. The dataset was taken from the Supplementary Materials of the authors’ work and consisted of 31,878 pairs of strains [8].

Additionally, we also conducted training and testing of advanced models of other researchers Qiato Jia [9] and Rui Yin [21] on our dataset from 2011 to 2024.

### 2.6. Statistical Analysis

To assess the uncertainty of the performance estimates, we calculated 95% confidence intervals for all reported metrics. For Accuracy, Sensitivity, and Specificity, which are proportions, we used the Wilson score interval. This method provides accurate coverage even for extreme proportions and moderate sample sizes.

For the Matthews Correlation Coefficient (MCC), which does not follow a simple binomial distribution, we employed the non-parametric bootstrap with 10,000 resamples [35]. For each test year and model configuration, bootstrap samples were generated by randomly sampling with replacement from the original set of strain pairs, and MCC was recalculated for each sample. The 95% confidence interval was then obtained as the 2.5th and 97.5th percentiles of the bootstrap distribution. All confidence intervals are reported in parentheses next to the point estimates in the results tables.

## 3. Results

### 3.1. Results of Model Calculations on Smith’s Quasi-Experimental Data

As an initial verification step and to establish a point of comparison with existing approaches documented in the literature, we first evaluated our model on the widely used quasi-experimental Smith dataset [8]. It is important to note that this dataset is characterized by well-separated antigenic clusters, and near-ceiling performance on it has become common for modern deep learning models. Therefore, our primary purpose here is not to claim superiority, but to confirm that our model operates within the expected performance range for this benchmark.

Calculations were performed on a two-layer convolutional model with an amino acid sequence length equal to the HA1 subunit (329), and they are presented in Table 5.

As can be seen from Table 5, our model achieved near-perfect metrics on this benchmark (Accuracy = 0.9996, MCC = 0.9964). While this confirms the model’s ability to learn from well-structured data, such results are expected given the high separability of the Smith dataset.

Table 6 shows the results of comparison of models in which colleagues used different versions of the Smith dataset (quasi-experimental Smith dataset) as input data, both complete and after certain filtering.

The comparison in Table 6 shows that our model’s performance on the full Smith dataset is consistent with, or slightly exceeds, the results reported by other groups. It is important to note, however, that direct comparisons are often complicated by differences in data preprocessing. For instance, Yuan Ling Xia et al. [23] applied filtering to remove pairs with a high number of substitutions, which may slightly lower the reported metrics. In contrast, we, like many other authors, opted to use the full, unfiltered set. Given that our model achieved near-perfect results, any filtering would likely result in only a marginal change in performance (e.g., a hypothetical reduction in the test set by 83% while retaining the single error would yield an accuracy of 0.9975).

In summary, the primary value of Smith’s dataset analysis is to demonstrate that our model is capable of learning antigenic relationships on a well-understood benchmark and to provide a reference point relative to prior work. The central focus of this study, however, remains the model’s performance under the more challenging and realistic time-split validation presented in the next section.

### 3.2. Results of Model Calculations on a Dataset from 2011 to 2024

In the next stage of our research, we decided to test our model in “real-world conditions,” training and testing it on the dataset prepared in this paper from 2011 to 2024 (strains dating back to 2007 were included).

Table 7 presents the performance of the two-layer and three-layer CNN models on the HA1 subunit for the test years 2022–2024, with 95% confidence intervals for all metrics. Complete results for the full HA sequence, as well as for the training and validation periods, are provided in Appendix A (Table A1, Table A2, Table A3 and Table A4).

For all versions of our model, a higher performance was recorded during the training and validation periods (Accuracy 0.84–0.90; MCC 0.65–0.78) than during the test periods (Accuracy 0.70–0.81; MCC 0.42–0.60). Moreover, the maximum values were observed in 2024, while the minimum was observed in 2023. As follows from the calculations provided, the performance of the models using both the entire hemagglutinin sequence and the HA1 subunit are not fundamentally different. This confirms the weak influence of the hemagglutinin stem portion on the HAI assay results.

Calculations also showed that introducing an additional convolutional layer into the model architecture did not improve its overall performance. The only exception was the 2023 test year, where the three-layer model showed a significant increase in the Matthews Convergence Metric (MCC): for the HA sequence from 0.417 to 0.479, and for the HA1 subunit sequence from 0.431 to 0.478.

To understand the reasons for this trend, we conducted an additional analysis, assessing the proportion of data within a distance of 1 log unit from the antigenic distance threshold (equal to 2) in both the entire dataset and in the test periods. As can be seen from Table 8, in all three test years (from 2022 to 2024), the proportion of such “difficult” data is statistically significantly higher than in the overall sample (*p* < 0.001). This means that the model worked with data closer to the threshold in the test periods than in training and validation. The least “difficult” year in terms of data was 2024, where the model showed the best result.

The higher performance for the 2022 test year compared to the same year in 2023 can be explained by the higher proportion of positive features in the 2023 test set (51.2% vs. 71.2%). However, despite the data imbalance, model performance in 2023 declined across all metrics (accuracy, sensitivity, specificity, MCC). This indicates that the observed degradation is not caused by feature imbalance. The simultaneous drop in sensitivity and specificity values likely indicates a decrease in separability between the positive and negative classes in the feature space, consistent with a conceptual drift scenario rather than a simple change in the data distribution. These results suggest that the third layer of the model allowed for the identification of higher-order patterns, thereby improving classification in 2023.

### 3.3. ROC Analysis and Threshold Sensitivity

To further evaluate the discriminative ability of our models and assess the robustness of the standard antigenic escape threshold (D = 2), we performed ROC analysis across a range of threshold values (D = 1.0 to 3.5) for all test years (2022–2024). It is important to emphasize that this analysis does not aim to identify an “optimal” threshold for our model; rather, it serves to demonstrate that model performance remains stable around the biologically grounded standard value of D = 2, which is based on the conventional four-fold reduction criterion in influenza surveillance [8,29].

**Figure 4:**
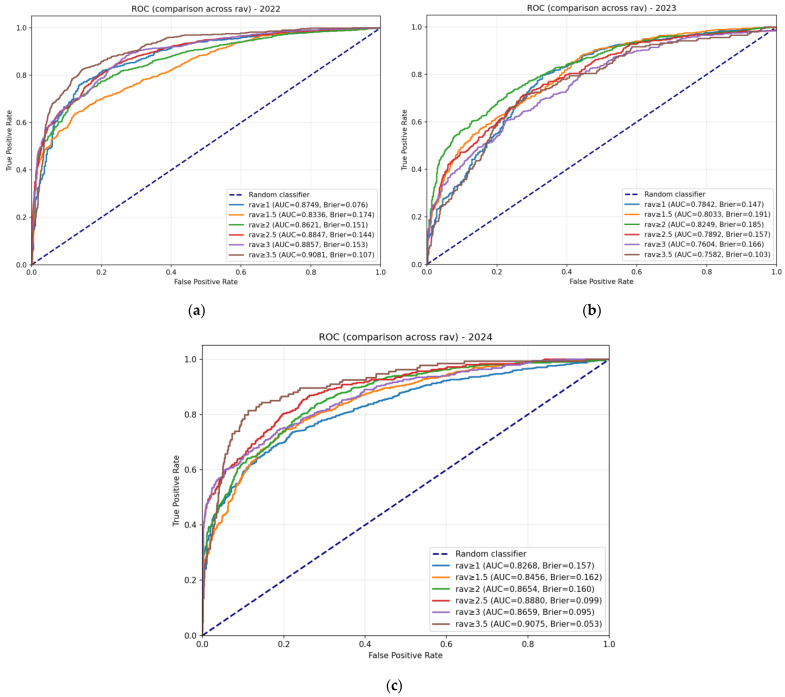
ROC curves for the three-layer subunit HA1 model across test years and thresholds. Panels show ROC curves for (**a**) 2022, (**b**) 2023, and (**c**) 2024 test years. Each panel displays curves for multiple antigenic escape thresholds (1.0 to 3.5, with step 0.5). The diagonal dashed line represents a random classifier.

**Figure A1:**
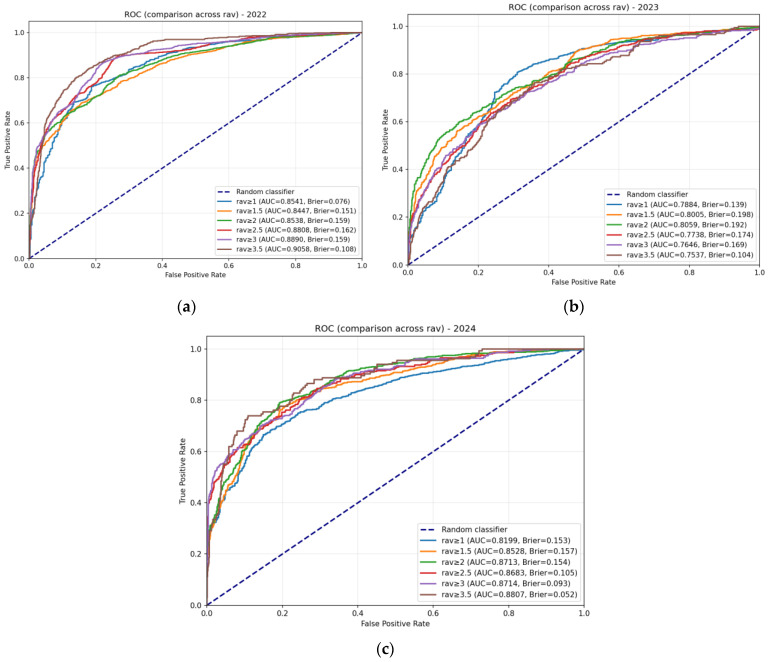
ROC curves for the two-layer subunit HA1 model across test years and thresholds. Panels show ROC curves for (**a**) 2022, (**b**) 2023, and (**c**) 2024 test years. Each panel displays curves for multiple antigenic escape thresholds (1.0 to 3.5, with step 0.5). The diagonal dashed line represents a random classifier.

**Figure A2:**
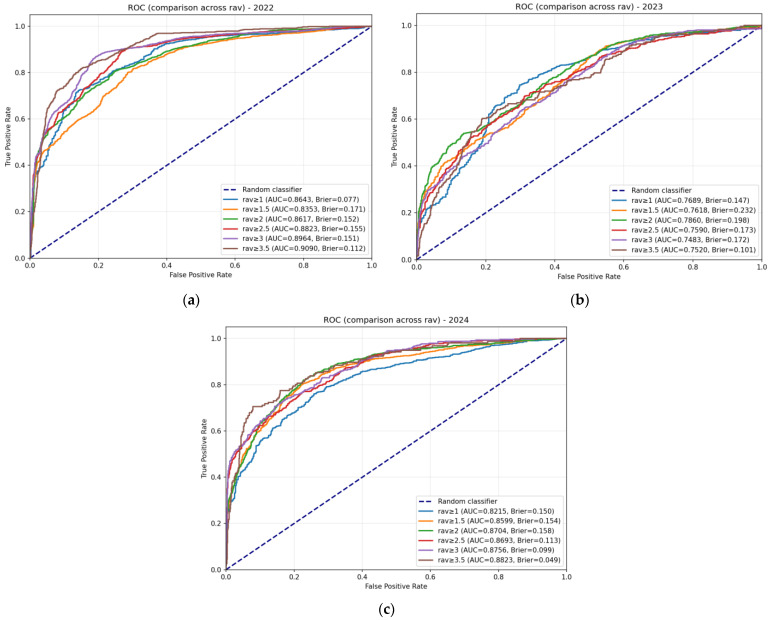
ROC curves for the two-layer HA model across test years and thresholds. Panels show ROC curves for (**a**) 2022, (**b**) 2023, and (**c**) 2024 test years. Each panel displays curves for multiple antigenic escape thresholds (1.0 to 3.5, with step 0.5). The diagonal dashed line represents a random classifier.

**Figure A3:**
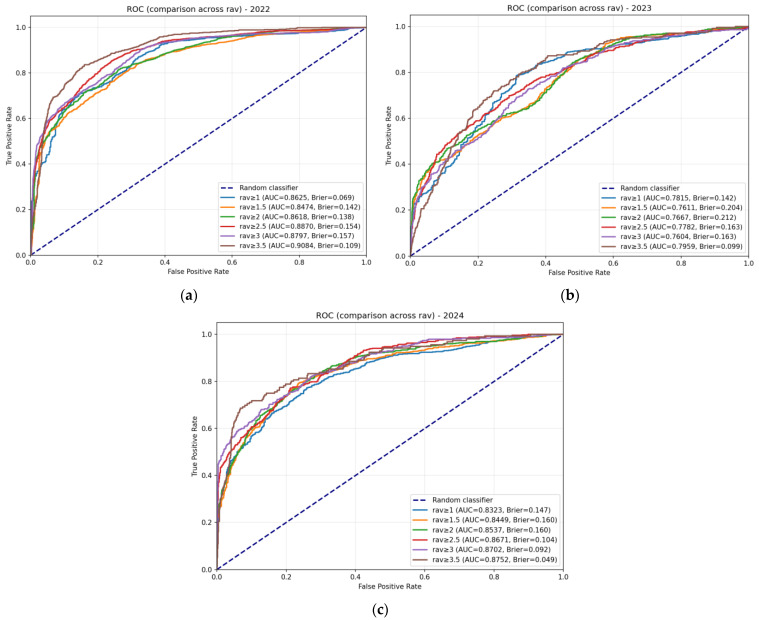
ROC curves for the three-layer HA model across test years and thresholds. Panels show ROC curves for (**a**) 2022, (**b**) 2023, and (**c**) 2024 test years. Each panel displays curves for multiple antigenic escape thresholds (1.0 to 3.5, with step 0.5). The diagonal dashed line represents a random classifier.

Figure 4 presents the ROC curves for the three-layer subunit HA1 model (which demonstrated the most robust performance in our comparative analysis) at multiple thresholds for each test year. Other ROC curves for both architectures (two-layer and three-layer) across all thresholds are provided in Appendix C (Figure A1, Figure A2 and Figure A3).

Table 9 summarizes the AUC values and Brier scores at the standard threshold equal to 2 for both architectures.

Several key observations emerge from the ROC analysis:

1. Strong discriminative ability at the standard threshold. Both architectures achieve AUC ≥ 0.805 for all test years at D = 2, with the highest values observed in 2024 (0.871 for two-layer, 0.865 for three-layer). These values confirm that the models effectively discriminate between antigenically similar and distinct strains.

2. Good calibration. Brier scores range from 0.151 to 0.192 at D = 2, indicating acceptable calibration across all models and years. The lowest scores (best calibration) are observed for the three-layer model in 2022 (0.151) and 2024 (0.160).

3. Robustness to threshold selection. Analysis of AUC values across all thresholds supports the robustness of our approach:

- Gradual, not abrupt, variation: AUC changes smoothly with threshold across all test years, with no evidence of sharp transitions that would indicate instability around the standard value D = 2.
- Consistent patterns across years: The relative difficulty of each test year (with 2023 being the most challenging, as documented in Table 7) is preserved across all threshold values, indicating that the observed performance differences reflect intrinsic characteristics of the data rather than artifacts of threshold selection.

These results confirm that while the standard threshold D = 2 is well supported by both biological convention and model performance, our findings are not sensitive to the exact choice of threshold. The model maintains a strong discriminative ability and good calibration across a wide range of threshold values.

### 3.4. Results of Comparison of the Developed Model with the Results of Advanced Models

Another way to evaluate our model is to compare its forecasts with the results of testing advanced models. The choice of models was determined by the technical feasibility of performing calculations with the dataset prepared as part of the current study.

Since in both works by Qiato Jia [9] and Rui Yin [21], the researchers used the Archetti–Horsfall distance as the antigenic distance and the calculations were performed with the HA1 sequence subunit, we used only symmetric data from our dataset (918 sequence pairs, 122 sequences) at the stage of data preparation for calculations using the MetaFluAD [9] and IAV-CNN models [21]. In addition, for training, validation, and testing, we selected a two-layer CNN with the HA1 subunit from our models and used our entire dataset. We also had to slightly modify the process of dividing the sample for training and testing in accordance with the calculations given in the above-mentioned works. As a result, we randomly divided the data from 2011 to 2024 into training (70%), validation (15%), and testing (15%) sets. The results are presented in Table 10.

The confidence intervals for test metrics in Table 10 reveal important insights about the comparison. Due to the limited test set size (138 symmetric pairs), the intervals for MetaFluad and IAV-CNN are 4.8–5.6 times wider than for our model (2731 pairs). The upper bounds of their test accuracy intervals (0.751 and 0.854) approach or exceed our point estimate (0.824), and the IAV-CNN interval (0.719–0.854) fully contains our model’s interval (0.810–0.838). This indicates that the lower point estimates for the comparator models may reflect, at least in part, the uncertainty inherent in small-sample evaluation rather than definitive performance differences. The advantage of our approach lies in its ability to utilize all available data for testing, providing more stable and reliable performance estimates.

## 4. Discussion

As part of a long-term research program aimed at improving vaccine strain selection, in this work, we developed and validated a convolutional neural network (CNN) for antigenic classification of influenza A(H3N2) virus. The core contribution of this study is the rigorous evaluation of the model under a temporally structured validation protocol, which simulates the key challenge of predicting antigenic properties for future epidemic seasons. The high performance observed on the Smith dataset serves primarily as a necessary sanity check, confirming the model’s basic functionality on a standard benchmark before its evaluation on the more challenging temporally structured data.

The model showed high results at the training and validation stages (Accuracy 0.84–0.90, MCC 0.65–0.78). However, testing on independent data from the last three years (2022–2024) showed statistically significant, but lower values in the range (Accuracy at 0.73–0.81, MCC 0.48–0.60, respectively). This is probably due to the fact that this year’s data is more concentrated near the threshold value of the antigenic distance compared to the training and validation periods.

Two model architectures with two and three convolutional layers were analyzed. Each was tested with both the full hemagglutinin (HA) length and the 1/HA1 subunit length. The results showed that model performance was not significantly affected by the input sequence length. Meanwhile, the three-layer architecture demonstrated qualitatively better results on the challenging 2023 dataset. These results suggest that the third convolutional layer of the model allowed for the detection of higher-order patterns, thereby improving classification in 2023.

The ROC analysis provides additional validation of our model’s performance and robustness. The consistently high AUC values (≥0.805 at the standard threshold D = 2) across all test years confirm the strong discriminative ability of both architectures, while the low Brier scores (≤0.192) indicate good calibration. The gradual variation in AUC across thresholds and the preservation of relative year-to-year difficulty patterns demonstrate that our conclusions are not sensitive to the exact choice of D = 2. This stability supports the use of the biologically standard threshold (four-fold titer reduction) within our modeling framework, and confirms that the performance fluctuations observed in 2023 reflect intrinsic characteristics of that season’s data rather than artifacts of threshold selection.

For additional validation, we compared the predictions of our model with the results of modern models, selecting those that were technically possible to run with our data. Following the methodology of the cited studies, we applied a random division of 2011–2024 data in the proportions of 70% (training), 15% (validation), and 15% (testing). Their training and validation results were comparable to ours, but they were lower on the test set. This decrease may be due to the small number of symmetric pairs of data for testing. It should be noted that this problem is typical for all models using the Archetti–Horsfall distance. Researchers usually bypass it by using data from several years for the test set, but this does not allow an adequate assessment of the suitability of the model for predicting the vaccine strain for the upcoming season. For example, in the qualitative work of Jing Meng et al. [22], the model showed a AUROC of 0.993 on validation; however, testing for individual years (2006–2022) gave a AUPRC range from 0.463 to 1, and testing was impossible for one of the years due to the lack of antigenically different pairs.

Thus, despite the formally high results of many models in “convenient” testing conditions, the task of increasing the performance of models in conditions close to the real forecast for the upcoming season remains relevant. In our opinion, the development of models for predicting the antigenicity of strains can proceed in two main directions:

1. A comprehensive consideration of the physicochemical consequences of amino acid substitutions in the hemagglutinin sequence, as well as accounting for its three-dimensional structure.

2. Accounting for the variability of the polyclonal immune response. A polyclonal set of antibodies produced in response to one strain often exhibits higher neutralizing activity against a certain heterologous strain than against a homologous one. This explains the fact that maximum titers in the HAI assay are often observed in reactions with heterologous strains. Models that take this feature into account [36,37] already exist, and their further development appears promising.

We also believe our overall approach is promising: integrating the cross-immunity model into a multi-strain epidemiological model will allow, on the one hand, for additional validation and improvement of the model using retrospective data, and on the other hand, for predicting the dominant strains of the next season and, therefore, more accurately determining the composition of effective vaccines.

## Figures and Tables

**Table 1:** Data characteristics.

(A) HA sequence			
Period	Number of unique sequence pairs/Proportion of positive attributes		
	Training	Validation	Testing
Training/Validation 2011–2021 Testing 2022	11469/50.2%	2875/49.3%	4112/51.2%
Training/Validation 2011–2022 Testing 2023	14758/50.3%	3698/50.3%	2026/71.2%
Training/Validation 2011–2023 Testing 2024	16378/52.4%	4104/52.3%	2126/77.0%
(B) subunit HA1 sequence			
Period	Number of unique sequence pairs/Proportion of positive attributes		
	Training	Validation	Testing
Training/Validation 2011–2021 Testing 2022	9242/49.7%	2318/49.5%	3281/51.8%
Training/Validation 2011–2022 Testing 2023	11866/50%	2975/50.5%	1656/70.5%
Training/Validation 2011–2023 Testing 2024	13190/52%	3307/52.6%	1710/76.4%

**Table 2:** Matrices selected from the AAindex1 database.

Hydrophobicity	Polarity	Volume	Accessible Surface	Charge
CIDH920104	GRAR740102	TSAJ990101	CHOC760101	KLEP840101
PONP800104	ZIMJ680103	COHE430101	CHOC760102	
WILM950101				
WILM950103				
WILM950104				

**Table 3:** Final list of matrices from the AAindex1 database.

Hydrophobicity	Polarity	Volume	Accessible Surface	Charge
WILM950101	ZIMJ680103	TSAJ990101	CHOC760102	KLEP840101

**Table 4:** CNN architectures for subunit HA1 sequence.

Component	2-conv CNN	3-conv CNN
Input tensor	(329, 2, 5)	(329, 2, 5)
Conv2D-1	filters = 320, kernel = 1 × 2, stride = 1, padding = same, activation = ReLU, dropout = 0.15	filters = 236, kernel = 1 × 2, stride = 1, padding = same, activation = ReLU, dropout = 0.15
Conv2D-2	filters = 196, kernel = 1 × 2, stride = 1, padding = same, activation = ReLU, dropout = 0.15	filters = 248, kernel = 3 × 1, stride = 1, padding = same, activation = ReLU, dropout = 0.15
Conv2D-3	—	filters = 32, kernel = 3 × 1, stride = 1, padding = same, activation = ReLU, dropout = 0.15
Pooling	None	None
Aggregation	Flatten	Flatten
Dense head	Dense(512) + ReLU + Dropout(0.35)	Dense(512) + ReLU + Dropout(0.3)
Output	Dense(1) + Sigmoid	Dense(1) + Sigmoid

**Table 5:** Data characteristics and the results of our model on Smith’s quasi-experimental data.

Validation		Evaluation Metrics				Testing		Evaluation Metrics			
Period	Number of Strain Pairs	Accuracy	Sensitivity	Specificity	MCC	Period	Number of Strain Pairs	Accuracy	Sensitivity	Specificity	MCC
1968–1998	6457	0.9984	0.9992	0.9935	0.9937	1999–2003	2366	0.9996	1	0.9933	0.9964

**Table 6:** Performance of various models on Smith’s quasi-experimental data.

Approaches	Evaluation Metrics			
	Accuracy	Sensitivity	Specificity	MCC
Multiple regression on physicochemical properties. Cui H., 2014 [26]	0.9696	0.9955	0.823	0.877
Decision tree. Huang J.-W., 2009 [25]	0.962	-	-	-
Joint random forest method. Yao Y., 2017 [18]	0.964	0.981	0.777	0.758
Stacked autoencoder. Tan Z., 2017 [27]	0.95	0.95	0.93	-
Deep learning approach. Xia Y.-L., 2021 [23]	0.9716	0.9685	0.9734	0.939
Our CNN	0.9996	1	0.9933	0.9964

**Table 7:** Performance metrics with 95% confidence intervals for test years 2022–2024 (subunit HA1 sequence).

Model	Two-Layer CNN			Three-Layer CNN		
Period	2022	2023	2024	2022	2023	2024
Accuracy (95%CI)	0.782 (0.768–0.796)	0.716 (0.694–0.737)	0.805 (0.786–0.823)	0.769 (0.754–0.783)	0.739 (0.717–0.760)	0.808 (0.789–0.826)
Sensitivity (95% CI)	0.821 (0.803–0.838)	0.714 (0.689–0.739)	0.691 (0.665–0.716)	0.754 (0.734–0.774)	0.722 (0.696–0.746)	0.753 (0.729–0.776)
Specificity (95% CI)	0.702 (0.680–0.723)	0.717 (0.679–0.752)	0.887 (0.859–0.911)	0.800 (0.781–0.818)	0.756 (0.719–0.790)	0.847 (0.816–0.874)
MCC (95% CI)	0.514 (0.485–0.543)	0.431 (0.387–0.474)	0.596 (0.562–0.629)	0.525 (0.496–0.554)	0.478 (0.435–0.520)	0.603 (0.568–0.636)

**Table 8:** The proportion of data within a distance of 1 log unit from the antigenic distance threshold.

Period	Number of Unique Sequence Pairs	Proportion of Data Within a Distance of 1 Log from the Threshold	*p*-Value
2011–2024	18,207	0.211	
2022	3281	0.297	<10^−16^
2023	1656	0.288	<10^−14^
2024	1710	0.247	<0.0002

**Table 9:** AUC and Brier scores at threshold equal 2 for both architectures.

Model	Two-Layer CNN			Three-Layer CNN		
Period	2022	2023	2024	2022	2023	2024
AUC	0.854	0.806	0.871	0.862	0.825	0.865
Brier Score	0.159	0.192	0.154	0.151	0.185	0.160

**Table 10:** Performance of various models on our dataset. For MetaFluad and IAV-CNN, results are based on symmetric pairs only (n_test = 138). For our model, results are based on the full dataset (n_test = 2731). Values in parentheses indicate 95% confidence intervals (Wilson score for Accuracy, Sensitivity, Specificity; bootstrap for MCC) for test metrics.

Approaches	Validation Model/Evaluation Metrics				Testing Model/Evaluation Metrics			
	Accuracy	Sensitivity	Specificity	MCC	Accuracy	Sensitivity	Specificity	MCC
Metafluad, Qitao Jia, 2024 [9]	0.825	0.799	0.866	0.651	0.678 (0.595–0.751)	0.578 (0.492–0.660)	0.756 (0.671–0.826)	0.339 (0.258–0.417)
IAV-CNN, Rui Yin, 2022 [21]	0.813	0.720	0.880	0.616	0.794 (0.719–0.854)	0.700 (0.616–0.773)	0.865 (0.793–0.915)	0.577 (0.503–0.646)
Our CNN	0.828	0.858	0.781	0.639	0.824 (0.810–0.838)	0.850 (0.836–0.864)	0.784 0.768–0.799)	0.633 (0.612–0.654)

## Data Availability

The original data and code presented in this study are openly available [https://github.com/xcom1994/flu_cnn, accessed on 7 February 2026]. These data were derived from the following resources available in the public domain [https://www.crick.ac.uk/research/platforms-and-facilities/worldwide-influenza-centre/annual-and-interim-reports, accessed on 7 February 2026], https://gisaid.org/resources/commentary-on-gisaid/, accessed on 7 February 2026]. The original contributions presented in this study are included in the article and Appendix A. Further inquiries can be directed to the corresponding author(s).

## Abbreviations

The following abbreviations are used in this manuscript:

MCC	Matthews Correlation Coefficient
CNN	Convolutional neural network
HAI	Hemagglutination inhibition
ICTV	International Committee on Taxonomy of Viruses
WHO	World Health Organization
PCA	Principal component analysis
GISAID	Global Initiative on Sharing All Influenza Data

## Appendix A

**Table A1:** Results obtained using a two-layer CNN (HA sequence).

Period	2011–2021		2011–2022		2011–2023	
Evaluation Metrics	Training	Validation	Training	Validation	Training	Validation
Accuracy	0.867	0.848	0.884	0.857	0.873	0.852
Sensitivity	0.918	0.902	0.920	0.901	0.907	0.890
Specificity	0.782	0.750	0.820	0.775	0.816	0.784
MCC	0.713	0.664	0.746	0.683	0.727	0.678
Period	Testing 2022		Testing 2023		Testing 2024	
Accuracy	0.797		0.707		0.806	
Sensitivity	0.831		0.678		0.716	
Specificity	0.723		0.738		0.872	
MCC	0.541		0.417		0.600	

**Table A2:** Results obtained using a three-layer CNN (HA sequence).

Period	2011–2021		2011–2022		2011–2023	
Evaluation Metrics	Training	Validation	Training	Validation	Training	Validation
Accuracy	0.879	0.851	0.888	0.848	0.868	0.848
Sensitivity	0.916	0.894	0.929	0.904	0.924	0.908
Specificity	0.816	0.773	0.814	0.744	0.774	0.741
MCC	0.739	0.673	0.753	0.661	0.714	0.665
Period	Testing 2022		Testing 2023		Testing 2024	
Accuracy	0.795		0.734		0.813	
Sensitivity	0.822		0.650		0.729	
Specificity	0.736		0.822		0.875	
MCC	0.542		0.479		0.615	

**Table A3:** Results obtained using a two-layer CNN (subunit HA1 sequence).

Period	2011–2021		2011–2022		2011–2023	
Evaluation Metrics	Training	Validation	Training	Validation	Training	Validation
Accuracy	0.865	0.851	0.869	0.846	0.858	0.839
Sensitivity	0.931	0.906	0.897	0.867	0.918	0.889
Specificity	0.753	0.758	0.821	0.808	0.758	0.759
MCC	0.708	0.677	0.717	0.670	0.692	0.655
Period	Testing 2022		Testing 2023		Testing 2024	
Accuracy	0.782		0.716		0.805	
Sensitivity	0.821		0.714		0.691	
Specificity	0.702		0.717		0.887	
MCC	0.514		0.431		0.596	

**Table A4:** Results obtained using a three-layer CNN (subunit HA1 sequence).

Period	2011–2021		2011–2022		2011–2023	
Evaluation Metrics	Training	Validation	Training	Validation	Training	Validation
Accuracy	0.885	0.856	0.899	0.854	0.876	0.852
Sensitivity	0.895	0.861	0.907	0.866	0.931	0.904
Specificity	0.868	0.848	0.884	0.835	0.785	0.766
MCC	0.756	0.698	0.784	0.691	0.733	0.681
Period	Testing 2022		Testing 2023		Testing 2024	
Accuracy	0.769		0.739		0.808	
Sensitivity	0.754		0.722		0.753	
Specificity	0.800		0.756		0.847	
MCC	0.525		0.478		0.603	

## Appendix B

**Table A5:** CNN architectures for HA sequence.

Component	2-conv CNN	3-conv CNN
Input tensor	(550, 2, 5)	(550, 2, 5)
Conv2D-1	filters = 320, kernel = 1 × 2, stride = 1, padding = same, activation = ReLU, dropout = 0.15	filters = 256, kernel = 1 × 2, stride = 1, padding = same, activation = ReLU, dropout = 0.1
Conv2D-2	filters = 196, kernel = 3 × 1, stride = 1, padding = same, activation = ReLU, dropout = 0.15	filters = 256, kernel = 3 × 1, stride = 1, padding = same, activation = ReLU, dropout = 0.2
Conv2D-3	—	filters = 64, kernel = 3 × 1, stride = 1, padding = same, activation = ReLU, dropout = 0.15
Pooling	None	None
Aggregation	Flatten	Flatten
Dense head	Dense(512) + ReLU + Dropout(0.35)	Dense(512) + ReLU + Dropout(0.35)
Output	Dense(1) + Sigmoid	Dense(1) + Sigmoid
